# The hemorrhagic transformation index score: a prediction tool in middle cerebral artery ischemic stroke

**DOI:** 10.1186/s12883-017-0958-3

**Published:** 2017-09-07

**Authors:** Mikhail N. Kalinin, Dina R. Khasanova, Murat M. Ibatullin

**Affiliations:** 1 Department of Neurology and Neurosurgery for Postgraduate Training, Kazan State Medical University, Kazan, Russia; 2 Department of Neurology, Interregional Clinical Diagnostic Center, 12A Karbyshev St, Kazan, 420101 Russia; 3 Department of Radiology, Interregional Clinical Diagnostic Center, Kazan, Russia

**Keywords:** Stroke, Middle cerebral artery, Complication, Hemorrhage, Prognosis, Hemorrhagic transformation

## Abstract

**Background:**

We aimed to develop a tool, the hemorrhagic transformation (HT) index (HTI), to predict any HT within 14 days after middle cerebral artery (MCA) stroke onset regardless of the intravenous recombinant tissue plasminogen activator (IV rtPA) use. That is especially important in the light of missing evidence-based data concerning the timing of anticoagulant resumption after stroke in patients with atrial fibrillation (AF).

**Methods:**

We retrospectively analyzed 783 consecutive MCA stroke patients. Clinical and brain imaging data at admission were recorded. A follow-up period was 2 weeks after admission. The patients were divided into derivation (DC) and validation (VC) cohorts by generating Bernoulli variates with probability parameter 0.7. Univariate/multivariate logistic regression, and factor analysis were used to extract independent predictors. Validation was performed with internal consistency reliability and receiver operating characteristic (ROC) analysis. Bootstrapping was used to reduce bias.

**Results:**

The HTI was composed of 4 items: Alberta Stroke Program Early CT score (ASPECTS), National Institutes of Health Stroke Scale (NIHSS), hyperdense MCA (HMCA) sign, and AF on electrocardiogram (ECG) at admission. According to the predicted probability (PP) range, scores were allocated to ASPECTS as follows: 10–7 = 0; 6–5 = 1; 4–3 = 2; 2–0 = 3; to NIHSS: 0–11 = 0; 12–17 = 1; 18–23 = 2; >23 = 3; to HMCA sign: yes = 1; to AF on ECG: yes = 1. The HTI score varied from 0 to 8. For each score, adjusted PP of any HT with 95% confidence intervals (CI) was as follows: 0 = 0.027 (0.011–0.042); 1 = 0.07 (0.043–0.098); 2 = 0.169 (0.125–0.213); 3 = 0.346 (0.275–0.417); 4 = 0.571 (0.474–0.668); 5 = 0.768 (0.676–0.861); 6 = 0.893 (0.829–0.957); 7 = 0.956 (0.92–0.992); 8 = 0.983 (0.965–1.0). The optimal cutpoint score to differentiate between HT-positive and negative groups was 2 (95% normal-based CI, 1–3) for the DC and VC alike. ROC area/sensitivity/specificity with 95% normal-based CI for the DC and VC were 0.85 (0.82–0.89)/0.82 (0.73–0.9)/0.89 (0.8–0.97) and 0.83 (0.78–0.88)/0.8 (0.66–0.94)/0.87 (0.73–1.0) respectively. McDonald’s categorical omega with 95% bias-corrected and accelerated CI for the DC and VC was 0.81 (0.77–0.84) and 0.82 (0.76–0.86) respectively.

**Conclusions:**

The HTI is a simple yet reliable tool to predict any HT within 2 weeks after MCA stroke onset regardless of the IV rtPA use.

## Background

Hemorrhagic transformation (HT), either asymptomatic (AHT) or symptomatic (SHT), is considered to be a notorious complication of acute ischemic stroke (AIS), associated with limited treatment options and long-term adverse outcomes [1]. It seems reasonable that efforts should be directed towards preventing HT before it occurs. Fortunately, it is more predictable than other types of intracranial hemorrhage.

In AIS patients, the incidence of HT induced by intravenous recombinant tissue plasminogen activator (IV rtPA) is reported to be 4.5–39.6% for AHT and 5.2–7.3% for SHT. In contrast, the rate of spontaneous AHT and SHT ranges from 13% to 43% and from 0.6% to 20% respectively [2, 3]. Although the proportion of AIS patients treated with IV rtPA is relatively small (4.7–21.4%) [4], the majority of authors have focused on searching HT predictors coupled with IV rtPA over the past decade. As a result, a variety of predictive clinical scores have emerged [5–9].

On the other hand, there is a lack of tools for making an accurate HT prediction in AIS patients who are not eligible for IV rtPA. That is especially important in the light of missing evidence-based data concerning the timing of anticoagulant resumption after AIS in patients with atrial fibrillation (AF). Recommendations on the initiation of anticoagulation are currently based on consensus opinion, in what is known as the “1–3–6–12 day rule” [10]. Therefore, the two-week timeframe following the AIS onset is the most critical for developing HT. In this instance, an accurate prediction of HT could make a difference in decision making to reinstitute anticoagulation. The middle cerebral artery (MCA) is by far the largest cerebral artery and is the vessel most commonly affected by cerebrovascular accident.

Given the background, we aimed to develop a simple and yet reliable instrument called the hemorrhagic transformation index (HTI) to predict any HT within 14 days after AIS onset in the MCA territory regardless of the use of IV rtPA.

## Methods

### Patients

Using prospectively collected clinical and radiological databases, we retrospectively identified 783 consecutive patients with AIS in the MCA territory who were admitted to the stroke unit of the Interregional Clinical Diagnostic Center, Kazan, Russia, within 12 h after onset between January 2013 and May 2016. The exclusion criteria were: involvement of other vascular territories; AIS following any surgery or endovascular procedure within 1 month; brain ischemic lesions due to an intracranial tumor, infection, cerebral venous thrombosis, subarachnoid hemorrhage, and arteriovenous malformation/fistula. In total, 1361 AIS patients were admitted over the specified period. The sample was drawn from the local Caucasian population.

The eligible patients received diagnostic tests and treatment in accordance with current national stroke guidelines. The permissible hospital length of stay was at least 14 days, which was determined by the state mandatory medical insurance standard for AIS patients.

Clinical baseline variables, including age, sex, risk factors, pre-admission medication, stroke subtype according to the Trial of ORG 10172 in Acute Stroke Treatment classification, NIHSS score, vital signs, blood tests, electrocardiogram (ECG), echocardiogram, and chest X-ray findings at admission were extracted from the medical charts. The NIHSS score was routinely and systematically assessed by neurologists. The time of AIS onset was documented as described by the patient or witness; if unknown, it was considered to be the last time the patient was seen well. In-hospital antithrombotic medication was logged for 14 consecutive days; the log was withdrawn earlier if HT occurred.

### Imaging protocol

Brain non-contrast computed tomography (CT) was performed using a multidetector CT scanner (Aquilion 64; Toshiba Medical Systems, Otawara, Japan). All CT scans were obtained with 0.5 mm slice thickness; the technical parameters were as follows: 120 kVp, 300 mA, rotation time 0.75 s, matrix size 512 × 512, helical scan mode, total scan time 9.7 s, reconstruction interval 5 mm. Window levels and widths were optimized for gray/white matter distinction. The Alberta Stroke Program Early CT score (ASPECTS), hyperdense MCA (HMCA) sign, and leukoaraiosis were routinely and systematically recorded at admission by radiologists. The HMCA sign was assessed by measurements of absolute attenuation of the affected and normal vessels. Absolute density of the affected MCA of >43 Hounsfield units and the MCA ratio of >1.2 on a non-contrast CT scan were regarded as the positive HMCA sign [11]. Diffuse hypodense areas involving the periventricular and/or centrum semiovale white matter were considered as leukoaraiosis. A follow-up CT scan was routinely done on hospitalization day 7 and 14 or at any time if required by a treating neurologist. All patients had at least one follow-up CT scan.

### Outcome measures

The outcome was retrospectively revised based on prospectively collected data. Any HT on a follow-up CT scan within 14 days after AIS onset was taken into account. A hemorrhage was considered symptomatic if it was not seen on a previous CT scan and there had subsequently been either a suspicion of hemorrhage or any decline in neurologic status [12]. According to the ECASS I trial [13], HT was further classified into hemorrhagic infarction type 1 (HI-1), or type 2 (HI-2), or parenchymal hematoma type 1 (PH-1), or type 2 (PH-2).

### Statistical analysis

Multinomial logistic regression with relative risk ratio (RRR) estimation was used to highlight the AHT and SHT association with poor outcomes (death, malignant edema, and dependency defined as the modified Rankin scale of >2 at discharge); the baseline category was the HT-negative group.

The intraclass correlation coefficient (ICC) was computed to assess inter-rater agreement for the ASPECTS and NIHSS. In order to calculate the ICC for the NIHSS, the same set of video files with NIHSS examination from six patients with different stroke severity was demonstrated to seven neurologists who regularly admitted patients to our stroke unit. The ICC for the ASPECTS was obtained in the similar manner: the identical pool of brain non-contrast CT scans from 33 patients with different ischemic burden was presented to four radiologists, who regularly evaluated brain CT scans at admission. Each doctor was evaluated separately and independently; the NIHSS and ASPECTS reference manuals were available on request. He or she had a chance to make any corrections during the evaluation process, but was not allowed to do so after his or her assessment had been completed.

A preliminary data analysis showed that 14 variables in 39.21% of observations were missing (Table 1).

**Table 1:** Variables with Missing Values

	Missing, *n* (%)	Valid, *n*
Cholesterol, total	107 (13.7)	676
LVEF	80 (10.2)	703
AST	61 (7.8)	722
Bilirubin, total	55 (7)	728
Fibrinogen	41 (5.2)	742
Albumin	37 (4.7)	746
Protein, total	18 (2.3)	765
Sodium	11 (1.4)	772
Potassium	10 (1.3)	773
Prothrombin time	5 (0.6)	778
ALT	4 (0.5)	779
Urea	2 (0.3)	781
Hematocrit	2 (0.3)	781
Hemoglobin	1 (0.1)	782

*Note*: *ALT* stands for Alanine transaminase, *AST* Aspartate transaminase, and *LVEF* Left ventricular ejection fraction (echocardiography, Simpson method)

The data were not missing completely at random (Little’s test: χ^2^(1706), 2336.69; *p* < 0.001). However, the omissions did occur accidentally because some tests were not available at the time of patient’s admission or the results were lost. Moreover, the missing variables correlated with other collected data. Therefore, it was reasonable to assume that the data were missing at random and multiple imputation (MI) was an appropriate technique to manage the absent values (Table 2).

**Table 2:** MI Specification

Software package	IBM SPSS Statistics, v.24; Armonk, NY
Random number generator	Mersenne twister
Imputation method	Fully conditional specification (MCMC)
Model type	Linear regression, no interaction terms
Number of iterations	100
Predictors	All collected data, including missing variables
Restrictions	Extrema of source data
Number of imputations	15
Quality of MI	No pattern on MCMC convergence charts FMI, RIV, RE (Table 4)

*Note*: *FMI* stands for Fraction of missing information, *MCMC* Markov chain Monte Carlo, *RE* Relative efficiency, and *RIV* Relative increase in variance

After obtaining the imputed data, the observations were divided into derivation (DC) and validation (VC) cohorts by generating Bernoulli variates with probability parameter 0.7.

Descriptive statistics included median with interquartile range (IQR) and percentage for continuous (the distribution was not normal) and categorical data respectively. The NIHSS and ASPECTS were treated as continuous variables because of multiple categories. Lists of univariate and multivariate HT predictors were obtained by fitting a binary logistic regression (BLR) model. Variables with univariate *p*-value ≤0.25 were further included in multivariate analysis, whereas only items with *p*-value <0.05 were kept in the multivariate BLR equation. Once the list of HT predictors was obtained by fitting a multivariate BLR model, we dropped the MI dataset because the included variables had no missing values in the source data.

In order to proceed with exploratory (EFA) and confirmatory (CFA) factor analysis, the Bartlett’s test of sphericity, Kaiser-Meyer-Olkin measure, and Doornik-Hansen test were carried out to check for patterned relationships between the HTI items, data sufficiency, and multivariate normality respectively. EFA by means of principal factor (PF) and principal component factor (PCF) techniques was performed with varimax and promax rotations to assess dimensionality of the HTI items and to extract variables with shared variance; an eigenvalue cut-off was 1.0. CFA with maximum likelihood estimation was applied to select the final model; goodness of fit was assessed with the Satorra-Bentler scaled χ^2^ test to adjust for data non-normality.

HTI internal consistency reliability (ICR) was evaluated with the ordinal α, Guttman λ_2_ and λ_4_ bounds, Raykov’s ρ, McDonald’s ω, and greatest lower bound [14, 15]. The values ≥0.7 were considered to be reliable. As the model included ordinal and dichotomous variables, a polychoric correlation matrix was used for EFA and ICR analysis except for McDonald’s categorical ω. The latter was computed by using the Green and Yang method [16].

The DC and VC were compared by using the Mann-Whitney U and Pearson χ^2^ tests for continuous and categorical variables respectively. The equality of kernel density estimate (KDE) for predicted probability (PP) of any HT between the multivariate BLR model and HTI score as well as for HTI scores between the DC and VC was evaluated with the two-sample Kolmogorov-Smirnov test.

Receiver operating characteristic (ROC) analysis was conducted to assess prognostic performance. The optimal cutpoint score to distinguish between HT-positive and negative groups was defined with the Youden index. Based on the VC appraisal, the predictive ability of the HTI was compared with several alternative tools by testing the area under the ROC curve (AUC) of each score against the HTI one. For each comparison, the Šidák-adjusted *p*-value was reported. The AUC equality was evaluated by using the DeLong algorithm [17].

Whenever possible, bootstrapping was performed with 1000 samples and computing either adjusted for ties bias-corrected and accelerated (BCa) or normal-based (NB) confidence intervals (CI) to reduce sampling bias, overfitting, and prediction errors.

## Results

Overall, HT occurred in 186 (23.8%) out of 783 cases, whereas SHT was determined in 98 (12.5%). HI-1 was established in 26 (3.3%); HI-2, 120 (15.3%); PH-1, 13 (1.7%); and PH-2, 27 (3.4%) observations. Patients with any HT were more likely to have a poor outcome: death (AHT: RRR, 4.8; 95% CI, 2.2–10.6; *p* < 0.001; SHT: RRR, 11.7; 95% CI, 6–22.6; *p* < 0.001), malignant edema (AHT: RRR, 16.7; 95% CI, 5.6–49.2; *p* < 0.001; SHT: RRR, 52.1; 95% CI, 19.6–138.9; *p* < 0.001), and dependency (AHT: RRR, 4.4; 95% CI, 2.5–7.7; *p* < 0.001; SHT: RRR, 30.4; 95% CI, 9.5–97.1; *p* < 0.001).

There was no difference between AHT and SHT groups with regard to timing (*p* = 0.08): the former, median day 2 (IQR, 1–4); the latter, median day 2 (IQR, 1–3). The median AIS onset time was 6 h (IQR, 2–11). Just under half of all cases (346; 44.2%) were admitted within the 4.5-h therapeutic window; in total, 67 (8.6%) patients were treated with IV rtPA.

The ICC was 0.95 (95% CI, 0.85–0.99; *p* < 0.001) and 0.78 (95% CI, 0.59–0.88; *p* < 0.001) for the NIHSS and ASPECTS respectively, which indicated excellent inter-rater agreement. There was no difference between the DC and VC (Table 3).

**Table 3:** Baseline Characteristics in the DC and VC

	DC (*n* = 535)	VC (*n* = 248)	*p*-Value
Clinical data, median (IQR)			
Age (y)	71 (60–78)	69 (61–77)	0.924
DBP (mm Hg)	90 (80–100)	100 (80–100)	0.025
Height (m)	1.65 (1.6–1.72)	1.65 (1.58–1.71)	0.063
Male sex, *n* (%)	289 (54)	125 (50.4)	0.346
NIHSS	8 (4–16)	8 (4–16)	0.574
Pulse rate (bpm)	78 (74–83)	78 (74–85)	0.155
SBP (mm Hg)	160 (140–180)	160 (140–180)	0.19
Time from onset (h)	5.5 (2–11)	6 (2.5–11)	0.265
Temperature (°C)	36.6 (36.4–36.6)	36.6 (36.5–36.6)	0.194
Weight (kg)	78 (68–88)	76.7 (68–85)	0.476
Stroke cause, *n* (%)			
Large-artery atherosclerosis	212 (39.6)	109 (44)	0.252
Cardioembolism	225 (42.1)	101 (40.7)	0.725
Small-vessel occlusion	79 (14.8)	33 (13.3)	0.587
Other determined etiology	7 (1.3)	1 (0.4)	0.241
Undetermined etiology	12 (2.2)	4 (1.6)	0.562
Risk factors, *n* (%)			
Acute myocardial infarction	25 (4.7)	8 (3.2)	0.348
AF history	210 (39.3)	94 (37.9)	0.719
Alcohol abuse	62 (11.6)	29 (11.7)	0.966
Atherosclerosis	528 (98.7)	248 (100)	0.07
Bleeding history	41 (7.7)	27 (10.9)	0.136
Chronic heart failure	62 (11.6)	28 (11.3)	0.903
Chronic liver failure	5 (0.9)	7 (2.8)	0.045
Chronic renal failure	20 (3.7)	11 (4.4)	0.642
Coronary artery disease	191 (35.7)	89 (35.9)	0.96
Diabetes mellitus	142 (26.5)	69 (27.8)	0.707
Dyslipidemia	216 (40.4)	103 (41.5)	0.759
Hypertension	497 (92.9)	230 (92.7)	0.937
Malignancy	10 (1.9)	4 (1.6)	0.801
Seizures at onset	10 (1.9)	3 (1.2)	0.502
Previous TIA/stroke	221 (41.3)	116 (46.8)	0.151
Brain CT, *n* (%)			
ASPECTS, median (IQR)	8 (6–9)	8 (6–9)	0.708
HMCA sign	141 (26.4)	66 (26.6)	0.939
Leukoaraiosis	347 (64.9)	168 (67.7)	0.429
Left hemispheric stroke	286 (53.5)	122 (49.2)	0.266
Right hemispheric stroke	234 (43.7)	122 (49.2)	0.154
Bihemispheric stroke	15 (2.8)	4 (1.6)	0.314
Blood tests, median (IQR)			
Albumin (g/L), *n* = 513/233	41.1 (38.6–43.1)	40.7 (38.8–42.9)	0.612
ALT (IU/L), *n* = 531/248	20 (14–28)	19 (13.2–29.8)	0.48
AST (IU/L), *n* = 495/227	24 (19–31)	23 (19–32)	0.939
APTT (s)	32 (28.8–35.3)	31.9 (28.7–34.5)	0.299
Bilirubin, total (μmol/L), *n* = 497/231	11.5 (8.3–17)	11.5 (8–16.1)	0.699
Cholesterol, total (mmol/L), *n* = 467/209	5.1 (4.3–5.9)	5.2 (4.3–6.1)	0.286
Creatinine (μmol/L)	90.6 (79.3–106)	90 (77.4–108)	0.851
Fibrinogen (g/L), *n* = 502/240	3.2 (2.6–4)	3.2 (2.6–4.2)	0.985
Glucose (mmol/L)	6.8 (5.9–8.3)	6.8 (5.9–8.1)	0.998
Hematocrit, *n* = 534/247	0.43 (0.38–0.46)	0.42 (0.37–0.45)	0.08
Hemoglobin (g/L), *n* = 535/247	141 (128–153)	139 (124–150)	0.12
INR	1.04 (0.96–1.15)	1.05 (0.98–1.15)	0.361
Platelet count (×10^9^ cells/L)	245 (199–306)	249.5 (201–301)	0.865
Protein, total (g/L), *n* = 523/242	67.9 (64.4–71.7)	68.4 (64.7–72.7)	0.159
Potassium (mmol/L), *n* = 530/243	4 (3.7–4.3)	4 (3.7–4.3)	0.223
PT (s), *n* = 531/247	12 (10.9–14.8)	12 (11.2–15.3)	0.408
RBC (×10^12^ cells/L)	4.63 (4.28–4.97)	4.55 (4.14–4.93)	0.032
Sodium (mmol/L), *n* = 530/242	139.5 (138–141.3)	139.1 (137–141)	0.164
Urea (mmol/L), *n* = 533/248	6 (4.7–7.7)	5.7 (4.6–7.6)	0.403
WBC (×10^9^ cells/L)	7.9 (6.5–9.7)	7.6 (6.3–9.5)	0.285
ECG, *n* (%)			
AF rhythm	164 (30.7)	77 (31)	0.911
HR (bpm), median (IQR)	79 (67–91)	80 (68–96)	0.157
Normal ECG	26 (4.9)	10 (4)	0.607
Other ECG changes	259 (48.4)	121 (48.8)	0.921
LVEF (%), *n* = 486/217, median (IQR)	57 (50–60)	57 (50–61)	0.993
Chest X-ray, *n* (%)			
Aortic atherosclerosis	462 (86.4)	217 (87.5)	0.661
Cardiomegaly	401 (75)	207 (83.5)	0.008
Normal chest X-ray	28 (5.2)	13 (5.2)	0.996
Pleural effusion	62 (11.6)	20 (8.1)	0.134
Pneumonia	48 (9)	23 (9.3)	0.891
Pulmonary congestion	215 (40.2)	109 (44)	0.32
Antithrombotic medication, *n* (%)			
Anticoagulant	28 (5.2)	14 (5.6)	0.812
Antiplatelet	390 (73)	201 (81)	0.014
Anticoagulant + antiplatelet	66 (12.3)	17 (6.9)	0.02
IV rtPA	51 (9.5)	16 (6.5)	0.152
Outcome, *n* (%)			
Any HT	126 (23.6)	60 (24.2)	0.844
SHT	69 (12.9)	29 (11.7)	0.636
HI-1	22 (4.1)	4 (1.6)	0.069
HI-2	82 (15.3)	38 (15.3)	0.999
PH-1	9 (1.7)	4 (1.6)	0.944
PH-2	13 (2.4)	14 (5.6)	0.022
Death	34 (6.4)	19 (7.7)	0.499
Malignant cerebral edema	32 (6)	14 (5.6)	0.852
Dependency	325 (60.7)	147 (59.3)	0.695

*Note*: *APTT* stands for Activated partial thromboplastin time, *DBP* Diastolic blood pressure, *INR* International normalized ratio, *PT* Prothrombin time, *RBC* Red blood cells, *SBP* Systolic blood pressure, and *WBC* White blood cells

Univariate analysis was summarized in Table 4.

**Table 4:** Univariate Analysis in the DC Using the MI Dataset

	Any HT (*n* = 126)	No HT (*n* = 409)	OR (95% CI)	*p*-Value	FMI	RIV	RE
Clinical data, median (IQR)							
Age (y)	74 (62–79)	70 (60–77)	1.016 (0.998–1.034)	0.077	0	0	1
DBP (mm Hg)	90 (80–100)	90 (80–100)	0.992 (0.979–1.005)	0.217	0	0	1
Height (m)	1.65 (1.6–1.7)	1.66 (1.6–1.73)	0.983 (0.961–1.006)	0.138	0	0	1
Male sex, *n* (%)	60 (47.6)	229 (56)	0.715 (0.479–1.066)	0.1	0	0	1
NIHSS	20 (14–23)	6 (3–10)	1.253 (1.206–1.302)	<0.001	0	0	1
Pulse rate (bpm)	80 (74–88)	78 (72–80)	1.037 (1.021–1.053)	<0.001	0	0	1
SBP (mm Hg)	155 (140–180)	160 (140–179)	0.999 (0.992–1.006)	0.683	0	0	1
Temperature (°C)	36.6 (36.4–36.7)	36.6 (36.4–36.6)	1.658 (0.904–3.041)	0.102	0	0	1
Weight (kg)	76 (65–90)	79.5 (70–87)	0.998 (0.986–1.01)	0.759	0	0	1
Risk factors, *n* (%)							
Acute myocardial infarction	16 (12.6)	9 (2.2)	6.39 (2.75–14.851)	<0.001	0	0	1
AF history	81 (63.8)	129 (31.6)	3.808 (2.508–5.783)	<0.001	0	0	1
Alcohol abuse	11 (8.7)	51 (12.5)	0.664 (0.335–1.316)	0.241	0	0	1
Atherosclerosis	124 (97.6)	404 (99)	0.409 (0.09–1.853)	0.246	0	0	1
Bleeding history	9 (7.1)	32 (7.8)	1.116 (0.518–2.405)	0.78	0	0	1
Chronic heart failure	24 (18.4)	38 (9.3)	0.441 (0.253–0.768)	0.004	0	0	1
Chronic liver failure	1 (0.8)	4 (1)	0.802 (0.89–7.237)	0.844	0	0	1
Chronic renal failure	7 (5.5)	13 (3.2)	1.772 (0.691–4.543)	0.233	0	0	1
Coronary artery disease	56 (44.1)	135 (33.1)	1.595 (1.062–2.395)	0.024	0	0	1
Diabetes mellitus	39 (30.7)	103 (25.2)	1.312 (0.847–2.034)	0.224	0	0	1
Dyslipidemia	41 (32.3)	175 (42.9)	0.635 (0.417–0.967)	0.034	0	0	1
Hypertension	118 (92.9)	379 (92.9)	0.997 (0.459–2.166)	0.994	0	0	1
Malignancy	4 (3.1)	6 (1.5)	2.179 (0.605–7.846)	0.233	0	0	1
Previous TIA/stroke	51 (40.2)	170 (41.7)	0.939 (0.626–1.41)	0.763	0	0	1
Seizures at onset	0 (0)	10 (2.5)	–	0.075^a^	0	0	1
Brain CT, *n* (%)							
ASPECTS, median (IQR)	4 (1–6)	8 (7–9)	0.499 (0.44–0.567)	<0.001	0	0	1
HMCA sign	88 (69.3)	54 (13.2)	14.792 (9.213–23.749)	<0.001	0	0	1
Leukoaraiosis	87 (68.5)	260 (63.7)	1.238 (0.809–1.894)	0.325	0	0	1
Left hemispheric stroke	73 (58)	213 (52)	1.291 (0.863–1.931)	0.214	0	0	1
Right hemispheric stroke	53 (42)	181 (44.3)	0.898 (0.6–1.344)	0.602	0	0	1
Bihemispheric stroke	0 (0)	15 (3.7)	–	0.028^a^	0	0	1
Stroke cause, *n* (%)							
Large-artery atherosclerosis	41 (32.5)	171 (41.8)	0.671 (0.441–1.023)	0.064	0	0	1
Cardioembolism	79 (62.7)	146 (35.7)	3.028 (2.002–4.58)	<0.001	0	0	1
Small-vessel occlusion	0 (0)	79 (19.3)	–	<0.001^a^	0	0	1
Other determined etiology	1 (0.8)	6 (1.5)	0.537 (0.064–4.506)	0.567	0	0	1
Undetermined etiology	5 (4)	7 (1.7)	2.373 (0.74–7.612)	0.146	0	0	1
LVEF (%), *n* = 120/366, median (IQR)	55 (46.25–59)	58 (53–61.25)	0.957 (0.938–0.975)	<0.001	0.026	0.027	0.998
Chest X-ray, *n* (%)							
Aortic atherosclerosis	114 (89.8)	348 (85.3)	1.512 (0.801–2.85)	0.203	0	0	1
Cardiomegaly	109 (85.8)	292 (71.6)	2.406 (1.398–4.141)	0.002	0	0	1
Normal chest X-ray	3 (2.4)	25 (6.1)	0.371 (0.11–1.249)	0.109	0	0	1
Pleural effusion	25 (19.7)	37 (9.1)	2.458 (1.414–4.271)	0.001	0	0	1
Pneumonia	21 (16.5)	27 (6.6)	2.796 (1.52–5.143)	0.001	0	0	1
Pulmonary congestion	78 (61.4)	137 (33.6)	3.149 (2.085–4.755)	<0.001	0	0	1
ECG, *n* (%)							
AF rhythm	70 (55.1)	94 (23)	4.102 (2.699–6.236)	<0.001	0	0	1
HR (bpm), median (IQR)	90 (73–107)	75 (65.25–88)	1.034 (1.023–1.045)	<0.001	0	0	1
Normal ECG	3 (2.4)	23 (5.6)	0.405 (0.12–1.372)	0.146	0	0	1
Other changes	68 (53.5)	191 (46.8)	1.309 (0.878–1.952)	0.186	0	0	1
Blood tests, median (IQR)							
Albumin (g/L), *n* = 122/391	40.4 (38.4–42.8)	41.2 (38.7–43.2)	0.957 (0.905–1.012)	0.124	0.026	0.026	0.998
ALT (IU/L), *n* = 126/404	19 (15–29)	20 (14–27.5)	1.001 (0.992–1.01)	0.849	0.001	0.001	1
APPT (s)	31.7 (28.1–35.1)	32.2 (29–35.4)	0.993 (0.97–1.015)	0.52	0	0	1
AST (IU/L), *n* = 120/375	27 (20.4–32)	23 (18–30.3)	1.001 (0.995–1.008)	0.697	0.031	0.032	0.998
Bilirubin, total (μmol/L), *n* = 117/380	14.04 (9.3–19.6)	10.9 (8.1–15.4)	1.043 (1.018–1.068)	0.001	0.067	0.071	0.996
Creatinine (μmol/L)	89 (77.7–104.9)	91 (79.9–106.7)	0.998 (0.991–1.006)	0.638	0	0	1
Cholesterol, total (mmol/L), *n* = 113/354	4.8 (4–5.8)	5.2 (4.4–5.9)	0.805 (0.676–0.96)	0.016	0.038	0.039	0.998
Fibrinogen (g/L), *n* = 121/381	3.5 (2.7–4.4)	3.11 (2.6–3.9)	1.266 (1.087–1.475)	0.002	0.04	0.041	0.997
Glucose (mmol/L)	7.5 (6.4–9.7)	6.6 (5.8–7.9)	1.061 (1.009–1.117)	0.021	0	0	1
Hematocrit, *n* = 126/407	0.43 (0.38–0.46)	0.42 (0.38–0.46)	0.993 (0.958–1.029)	0.679	0	0	1
Hemoglobin (g/L)	141 (127–152)	141 (128.625–153)	0.997 (0.987–1.006)	0.515	0	0	1
INR	1.06 (0.98–1.15)	1.04 (0.95–1.12)	0.919 (0.467–1.808)	0.808	0	0	1
Platelet count (×10^9^ cells/L)	236 (186–286)	248 (206–309)	0.998 (0.995–1)	0.048	0	0	1
Potassium (mmol/L), *n* = 126/404	4 (3.7–4.3)	4 (3.6–4.2)	1.247 (0.862–1.802)	0.241	0.009	0.009	0.999
Protein, total (g/L), *n* = 126/397	67.6 (64–71.2)	68 (64.8–71.7)	0.987 (0.953–1.021)	0.444	0.006	0.006	1
PT (s), *n* = 126/405	12.2 (11.3–13.8)	11.8 (10.8–16.1)	0.976 (0.932–1.022)	0.305	0.002	0.002	1
RBC (×10^12^ cells/L)	4.64 (4.23–5.01)	4.63 (4.3–4.97)	0.941 (0.664–1.332)	0.73	0	0	1
Sodium (mmol/L), *n* = 126/404	139.3 (138–141.9)	139.5 (138–141.3)	1.015 (0.957–1.077)	0.624	0.011	0.011	0.999
Urea (mmol/L), *n* = 126/406	6.36 (5.2–8.5)	5.81 (4.5–7.3)	1.089 (1.02–1.161)	0.01	0	0	1
WBC (×10^9^ cells/L)	8.8 (6.6–11.4)	7.8 (6.5–9.3)	1.145 (1.071–1.225)	<0.001	0	0	1
Antithrombotic medication, *n* (%)							
Anticoagulant	5 (4)	23 (5.6)	0.693 (0.258–1.863)	0.468	0	0	1
Antiplatelet	68 (54)	322 (78.7)	0.317 (0.208–0.483)	<0.001	0	0	1
Anticoagulant + antiplatelet	29 (23)	37 (9.1)	3.006 (1.76–5.132)	<0.001	0	0	1
IV rtPA	24 (19)	27 (6.6)	3.329 (1.842–6.015)	<0.001	0	0	1

*Note*: ^a^Perfect predictor. Instead of BLR, Pearson χ^2^ test was used

### Multivariate analysis

Although univariate *p*-values for leukoaraiosis and international normalized ratio (INR) were above our acceptable threshold, we included them in the multivariate analysis because some authors had proposed them as risk factors [18, 19]. As a result of fitting a multivariate BLR model, seven variables were kept in the final equation (Table 5).

**Table 5:** Multivariate Analysis (DC, *n* = 535)

	Coefficient	Bias	Bootstrap SE	OR (95% BCa CI)	*p*-Value
ASPECTS	−0.472	−0.026	0.092	0.62 (0.52–0.75)	<0.001
AF on ECG	1.157	0.029	0.405	3.18 (1.47–6.66)	0.002
Male sex	0.88	0.055	0.381	2.41 (1.12–5.15)	0.027
NIHSS	0.135	0.004	0.029	1.15 (1.08–1.21)	<0.001
HR on ECG (bpm)	0.027	0.001	0.008	1.03 (1.01–1.04)	0.001
HMCA sign	1.041	0.001	0.346	2.83 (1.44–5.45)	0.002
INR	−3.304	−0.179	0.92	0.04 (0.01–0.22)	<0.001

*Note*: *SE* stands for Standard error

Swapping AF on ECG for the AF history variable increased the Bayesian and Akaike information criteria by 1.18, which slightly favored the initial model. Overall, the multivariate BLR model was statistically significant (Wald test: χ^2^(7), 87.76; *p* < 0.001; −2log-likelihood, 271.93; Cox-Snell pseudo-R^2^, 0.44; Nagelkerke pseudo-R^2^, 0.67). It explained variance of 78.4% (variance of latent variable, y* = 11.13; error, ε = 3.29) and fitted the data well (Hosmer-Lemeshow goodness of fit test for 10 groups: χ^2^(8), 6.87; *p* = 0.551). Neither significant interactions nor polynomial terms were found.

There was no specification error (Pregibon’s link test: linear predicted value, *p* < 0.001; linear predicted value squared, *p* = 0.54). Assumption of linearity between independent variables and log odds was confirmed by the LOWESS graph. Multicollinearity was not an issue: the extrema of the variance inflation factor were 1.01 and 2.29.

**Fig. 1:**
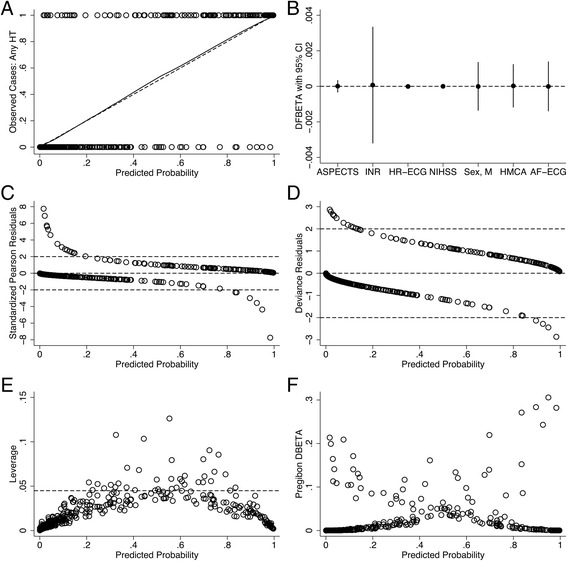
Multivariate BLR Model Diagnostics. **a** LOWESS graph, bandwidth = 0.8. Each hollow circle is an individual observation. **b** Influence of each individual observation on the coefficient estimate (dfbeta). **c** Standardized Pearson residuals. **d** Deviance residuals. **e** Leverage. The dashed line corresponds to the 3-fold leverage mean. **f** Pregibon’s influential statistics (dbeta)

Although standardized Pearson and deviance residuals exceeded 2 in a few observations, their leverage and Pregibon’s influential statistics (dbeta) turned out to be very small. Moreover, removing those observations did not significantly change the equation coefficients. Influence of each individual observation on the coefficient estimate (not adjusted for the covariate pattern), dfbeta, was not strong. However, the most sensitive was the INR variable (Fig. 1).

Model sensitivity, specificity, positive and negative predictive values were 76.2%, 95.6%, 84.2%, and 92.9% respectively. The model accurately classified 91% of the observations, whereas the equation without any independent variables classified correctly only 76.5% of the cases.

**Fig. 2:**
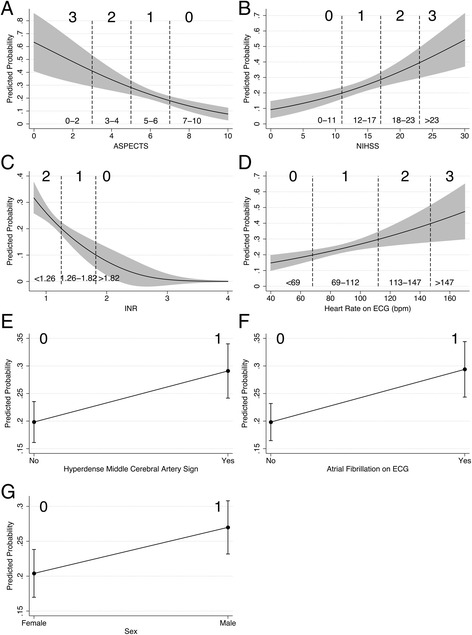
Derivation of HTI Score. **a** ASPECTS. **b** NIHSS. **c** INR. **d** HR on ECG. **e** HMCA sign. **f** AF on ECG. **g** Sex. Grey areas and capped lines represent 95% CI. Numbers at the top indicate HTI scores; at the bottom, units of variables. The confounders are held constant at their observed values

**Fig. 3:**
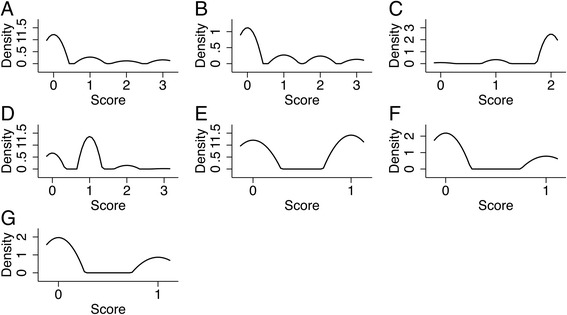
KDE for HTI Items. Kernel = Epanechnikov for all items. **a** ASPECTS; bandwidth = 0.1899. **b** NIHSS; bandwidth = 0.1899. **c** INR; bandwidth = 0.116. **d** HR on ECG; bandwidth = 0.1528. **e** Sex; bandwidth = 0.1278. **f** HMCA sign; bandwidth = 0.113. **g** AF on ECG; bandwidth = 0.1182

Based on PP tables and plots, we divided each predictor into categories and allocated them points according to the PP range in order to draw the HTI score. Given the Doornik-Hansen test (χ^2^(14), 3665.64; *p* < 0.001), the distribution of the newly derived HTI items was not multivariate normal (Table 6; Figs. 2 and 3).

**Table 6:** Derivation of the HTI Score

	PP Range^a^	Allocated Points
ASPECTS		
10–7	0.1–0.2	0
6–5	0.2–0.3	1
4–3	0.3–0.4	2
2–0	>0.4	3
NIHSS		
0–11	0.1–0.2	0
12–17	0.2–0.3	1
18–23	0.3–0.4	2
>23	>0.4	3
INR		
>1.82	<0.1	0
1.26–1.82	0.1–0.2	1
≤1.25	>0.2	2
HR on ECG (bpm)		
40–68	0.1–0.2	0
69–112	0.2–0.3	1
113–147	0.3–0.4	2
>147	>0.4	3
AF on ECG	0.2–0.3	1
HMCA sign	0.2–0.3	1
Male sex	0.2–0.3	1

*Note*: ^a^Holding all other variables constant at their observed values

### Factor analysis

**Fig. 4:**
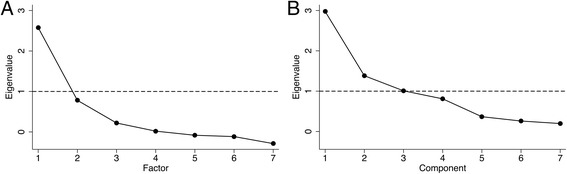
Scree Plot. EFA by using (**a**) PF and (**b**) PCF methods

The Bartlett’s test of sphericity (χ^2^(21), 721.74; *p* < 0.001) and the Kaiser-Meyer-Olkin measure of 0.71 demonstrated that the HTI items did have patterned relationships and were sufficient for EFA. PF EFA established a unidimensional scale, i.e. there was only one factor that explained a cumulative variance of 82.8%. By means of PCF EFA, the factor was discovered to consist of cerebral (ASPECTS, NIHSS, and HMCA sign) and extracerebral (resting heart rate (HR) on ECG, AF on ECG, sex, and INR) components as we called them. However, the resting HR on ECG, INR, and sex variables showed high uniqueness values and low factor loadings; therefore, we had to drop them. The final 4-item HTI was strongly supported by subsequent CFA (Tables 7 and 8; Fig. 4).

**Table 7:** Factor/Component Loadings and Uniqueness in EFA of the HTI Items

	No Rotation			Varimax Rotation		Promax Rotation		Uniqueness	
	F1	C1	C2	C1	C2	C1	C2	PF	PCF
ASPECTS	0.84	0.86		0.9		0.9		0.29	0.17
HR on ECG	0.34	0.43	−0.6		0.73		0.75	0.89	0.45
INR			0.56		−0.54		−0.58	0.99	0.68
NIHSS	0.83	0.86		0.89		0.89		0.32	0.18
Male sex	−0.33	−0.43			−0.4		−0.39	0.89	0.76
HMCA sign	0.81	0.84	0.36	0.91		0.91		0.35	0.18
AF on ECG	0.56	0.65	−0.59		0.83		0.82	0.69	0.23
Eigenvalue	2.58	2.98	1.38	NA	NA	NA	NA	NA	NA
Variance, %	82.8	42.5	19.76	37.56	24.7	39.33	28.1	NA	NA

*Note*: Empty cells indicate that absolute factor or component loading value is <0.3. *C1* stands for Component 1, *C2* Component 2, *F1* Factor 1, and *NA* Not applicable

**Table 8:** CFA. Goodness of Fit Statistics

	F1-V4	F1-V5	F1-V6	F1-V7	F2	Description
Likelihood ratio						
χ^2^(2/5/9/14/13)	0.5	56.17	87.26	103.66	18.19	Model vs. saturated
*p*-value	0.78	<0.001	<0.001	<0.001	0.15	
χ^2^(6/10/15/21/21)	594.14	664.18	711.01	727.4	727.4	Baseline vs. saturated
*p*-value	<0.001	<0.001	<0.001	<0.001	<0.001	
Satorra-Bentler test						
χ^2^(2/5/9/14/13)	0.4	50.96	82.63	100.31	18.08	Model vs. saturated
*p*-value	0.82	<0.001	<0.001	<0.001	0.16	
χ^2^(6/10/15/21/21)	475.75	574.14	649.06	684.79	684.79	Baseline vs. saturated
*p*-value	<0.001	<0.001	<0.001	<0.001	<0.001	
Population error						
RMSEA	<0.001	0.138	0.127	0.109	0.027	Root mean squared error of approximation
90% CI	0–0.056	0.107–0.172	0.104–0.153	0.09–0.13	0–0.054	
*p*-close	0.932	<0.001	<0.001	<0.001	0.909	Probability RMSEA ≤0.05
RMSEA-SB	<0.001	0.131	0.124	0.107	0.027	Satorra-Bentler RMSEA
Information criteria						
AIC	3726.82	4682.79	5446.18	6121.68	6038.21	Akaike information criterion
BIC	3778.21	4747.02	5523.26	6211.6	6132.42	Bayesian information criterion
Baseline comparison						
CFI	1	0.92	0.89	0.87	0.99	Comparative fit index
TLI	1	0.84	0.81	0.81	0.99	Tucker-Lewis index
CFI-SB	1	0.92	0.88	0.87	0.99	Satorra-Bentler CFI
TLI-SB	1	0.84	0.81	0.81	0.99	Satorra-Bentler TLI
Size of residuals						
SRMR	0.01	0.08	0.08	0.08	0.03	Standardized root mean squared residual
CD	0.83	0.83	0.83	0.83	0.96	Coefficient of determination

*Note*: *F1-V4* indicates one-factor model with 4 variables (ASPECTS, NIHSS, HMCA sign, and AF on ECG), *F1-V5* one-factor model with 5 variables (4 previous variables + HR on ECG), *F1-V6* one-factor model with 6 variables (5 previous variables + sex), *F1-V7* one-factor model with 7 variables (6 previous variables + INR), and *F2* two-factor model with factor 1 (ASPECTS, NIHSS, and HMCA sign) and factor 2 (AF on ECG, INR, sex, and HR on ECG)

**Fig. 5:**
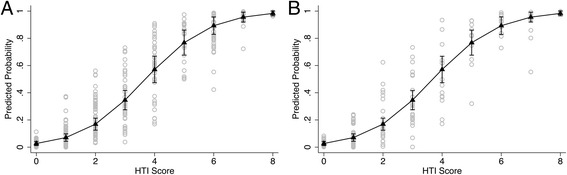
Calibration Plot. The scatterplots display PP of any HT for individual observations obtained from multivariate analysis (Table 5) and arranged by the HTI score: (**a**) The DC, (**b**) The VC. The superimposed connected line graph drawn from the DC demonstrates PP of any HT for each HTI score with 95% CI adjusted for sex, INR, and HR on ECG (Table 11). The confounders are held constant at their observed values

Once the HTI was definitively established, crude PP of any HT was computed for each score by using BLR. Although the dropped items were no longer a part of the HTI, we put them into the BLR equation for confounding adjustment. Unsurprisingly, the confounders exerted only a minor influence on the overall HTI OR by increasing it up to 12% and had no significant effect on the OR of the separate HTI items (Tables 9, 10 and 11; Fig. 5).

**Table 9:** BLR Coefficients, Bias, and Bootstrap SE for the HTI (DC, *n* = 535)

	Crude			Adjusted^a^		
	Coefficient	Bias	Bootstrap SE	Coefficient	Bias	Bootstrap SE
HTI, overall	0.941	0.012	0.085	1.03	0.027	0.109
HTI items						
ASPECTS	0.815	0.031	0.165	0.914	0.047	0.188
NIHSS	0.958	0.02	0.175	1.053	0.038	0.201
HMCA sign	1.157	−0.005	0.336	1.214	0.008	0.347
AF on ECG	1.115	0.002	0.317	1.214	0.031	0.403

*Note*: ^a^Confounders (sex, INR, and HR on ECG) were measured in allocated points (Table 6)

**Table 10:** OR for the HTI (DC, *n* = 535)

	Crude		Adjusted^a^		Crude vs. Adjusted^b^	
	OR (95% BCa CI)	*p*-Value	OR (95% BCa CI)	*p*-Value	χ^2^(1)	*p*-Value
HTI, overall	2.56 (2.19–3.02)	<0.001	2.80 (2.32–3.39)	<0.001	4.03	0.045
HTI items						
ASPECTS	2.26 (1.64–3.07)	<0.001	2.49 (1.75–3.54)	<0.001	2.4	0.121
NIHSS	2.61 (1.88–3.7)	<0.001	2.87 (1.91–4.15)	<0.001	1.91	0.167
HMCA sign	3.18 (1.71–6.24)	0.001	3.37 (1.73–6.89)	<0.001	0.33	0.565
AF on ECG	3.05 (1.71–5.88)	<0.001	3.37 (1.55–7.4)	0.003	0.27	0.605

*Note*: ^a^Confounders (sex, INR, and HR on ECG) were measured in allocated points (Table 6). ^b^Wald test was performed

**Table 11:** PP of Any HT for Each HTI Score

HTI Score	Crude		Adjusted^a^	
	PP (95% CI)	*p*-Value	PP (95% CI)	*p*-Value
0	0.03 (0.015–0.044)	<0.001	0.027 (0.011–0.042)	0.001
1	0.073 (0.047–0.099)	<0.001	0.07 (0.043–0.098)	<0.001
2	0.168 (0.126–0.21)	<0.001	0.169 (0.125–0.213)	<0.001
3	0.34 (0.274–0.407)	<0.001	0.346 (0.275–0.417)	<0.001
4	0.569 (0.479–0.66)	<0.001	0.571 (0.474–0.668)	<0.001
5	0.772 (0.686–0.858)	<0.001	0.768 (0.676–0.861)	<0.001
6	0.897 (0.838–0.955)	<0.001	0.893 (0.829–0.957)	<0.001
7	0.957 (0.925–0.989)	<0.001	0.956 (0.92–0.992)	<0.001
8	0.983 (0.967–0.998)	<0.001	0.983 (0.965–1.0)	<0.001

*Note*: ^a^Confounders (sex, INR, and HR on ECG) were held constant at their observed values

**Fig. 6:**
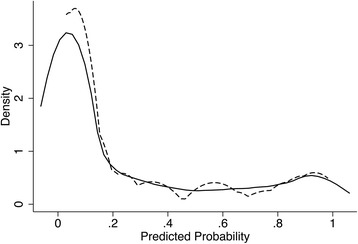
KDE for PP of Any HT. Kernel = Epanechnikov, bandwidth = 0.0623. The solid line represents the multivariate BLR model (Table 5); the dashed line, the HTI score (Table 11)

KDE for PP of any HT was equal between the multivariate BLR model and HTI score (D = 0.184; *p* = 0.371). Thus, the HTI score was considered as a surrogate for the multivariate BLR model (Fig. 6).

### ICR and ROC analysis

**Fig. 7:**
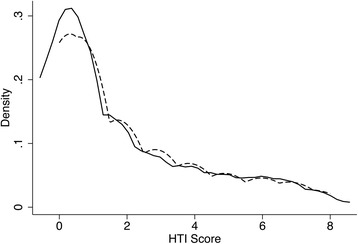
KDE for HTI Scores. Kernel = Epanechnikov, bandwidth = 0.5697. The solid line represents the DC; the dashed line, the VC

Given multiple reliability tests, HTI ICR was considered to be fair enough for the DC and VC alike. There was also no difference in the AUC (χ^2^(1), 0.01; *p* = 0.93) and KDE for the HTI scores (D = 0.02; *p* = 1.0) between both cohorts (Table 12; Fig. 7).

**Table 12:** HTI ICR and ROC Analysis

	DC, *n* = 535	VC, *n* = 248
Ordinal α	0.82	0.83
Guttman bounds		
λ_2_	0.83	0.85
λ_4_	0.84	0.88
Raykov’s ρ	0.81	0.81
McDonald’s ω		
Categorical (95% BCa CI)	0.81 (0.77–0.84)	0.82 (0.76–0.86)
Hierarchical	0.83	0.87
Total	0.89	0.91
Greatest lower bound	0.84	0.89
ROC analysis (95% NB CI)		
AUC	0.85 (0.82–0.89)	0.83 (0.78–0.88)
Youden index	0.7 (0.63–0.78)	0.67 (0.57–0.77)
Cutpoint	2 (1–3)	2 (1–3)
Sensitivity	0.82 (0.73–0.9)	0.8 (0.66–0.94)
Specificity	0.89 (0.8–0.97)	0.87 (0.73–1.0)

Taking into account that alternative scores had been developed in AIS patients with slightly different clinical settings, the HTI prognostic performance was considered to be at least non-inferior to the competitors (Tables 13 and 14).

**Table 13:** Comparison of Different Composite Scores for Predicting HT in AIS Patients

Score	Predictors		Sample Size	Cerebral Vascular Territory	Predicted Outcome	IV rtPA Settings
Cucchiara [6]	Clinical	Age, NIHSS	1205	Any	Any HT	Yes
	Laboratory	Glucose, platelet count				
GRASPS [9]	Clinical	Age, ethnicity, NIHSS, sex, SBP	10,242	Any	SHT	Yes
	Laboratory	Glucose				
HAS-BLED [46]	Clinical	Age, alcohol abuse, bleeding history, drugs, SBP, stroke/TIA	3978	Any	Major Bleeding^a^	No
	Laboratory	Liver and renal function tests, INR				
HAT [5]	Clinical	Diabetes mellitus, NIHSS	400	Any	SHT	Yes
	Imaging	ASPECTS				
	Laboratory	Glucose				
HeRS [31]	Clinical	Age	345	Any	Any HT	Regardless
	Imaging	Infarct volume				
	Laboratory	Estimated glomerular filtration rate				
HTI	Clinical	NIHSS	783	MCA	Any HT	Regardless
	Imaging	ASPECTS, HMCA sign				
	Laboratory	AF on ECG				
iScore [32]	Clinical	AF, age, cancer, chronic heart failure, NIHSS, preadmission disability, renal dialysis, sex, stroke subtype	1696	Any	Any HT	Yes
	Laboratory	Glucose				
SEDAN [8]	Clinical	Age, NIHSS	1802	Any	SHT	Yes
	Imaging	ASPECTS, HMCA sign				
	Laboratory	Glucose				
SITS-SICH [7]	Clinical	Age, antiplatelet medication, hypertension, NIHSS, stroke onset to treatment time, SBP, weight	31,627	Any	SHT	Yes
	Laboratory	Glucose				
SPAN-100 [33]	Clinical	Age, NIHSS	624	Any	Any HT	Regardless

*Note*: ^a^1-year risk for major bleeding (intracranial, hospitalization, hemoglobin decrease > 2 g/L, and/or transfusion) in a cohort of real-world patients with AF

**Table 14:** AUC Comparison Between the HTI and Alternative Scores (VC, *n* = 248)

Score	AUC (95% NB CI)	χ^2^(1)	*p*-Value
HTI	0.83 (0.78–0.88)	–	–
Cucchiara	0.72 (0.67–0.77)	31.6	<0.001
GRASPS	0.80 (0.75–0.84)	10.98	<0.001
HAS-BLED	0.53 (0.47–0.59)	86.47	<0.001
HAT	0.82 (0.77–0.88)	4.34	0.037
HeRS^a^	0.81 (0.75–0.86)	4.11	0.043
iScore	0.83 (0.79–0.87)	2.93	0.087
SEDAN	0.82 (0.76–0.87)	11.59	<0.001
SITS-SICH^b^	0.72 (0.66–0.78)	25.06	<0.001
SPAN-100	0.59 (0.53–0.65)	111.26	<0.001

*Note*: ^a^Infarct volume was calculated on follow-up CT scans (≥12 h after the initial imaging) by using the ABC/2 formula. ^b^For patients, who were not eligible for IV rtPA, stroke onset to treatment time was considered as stroke onset to admission time

## Discussion

The incidence of HT showed in our study echoes the rate reported in literature. We have also reaffirmed the concept that AHT is not clinically innocuous. The study unequivocally reiterates infarct size, stroke severity, large-artery occlusion, and cardioembolism defined by ASPECTS, NIHSS, HMCA sign, and AF respectively are well-established independent HT predictors [20, 21]. In our HTI score, we use the presence of AF on ECG at admission rather than AF history for the reason discussed in the Results. However, other known predictors – platelet count, cholesterol level, age, hypertension, renal failure, hyperglycemia, and leukoaraiosis – have shown no independent association with any HT in our cohort; similar results were obtained by other authors [22–26]. The mechanism of this association needs to be explored.

Although women tend to be more likely HT-positive in our univariate BLR model, multivariate analysis reveals the opposite. It seems there is still controversy about the sex propensity for developing HT [27].

Accelerated HR at rest is known to be associated with an increased risk of stroke especially recurrent [28]. Since AIS commonly induces change in cardiovascular responses, post-stroke HR at admission could be a potential marker to identify patients at risk for short-term deterioration and long-term poor outcomes [29]. To the best of our knowledge, we have not found any literature, concerning HR correlations with HT. Here, we report that the more the HR is accelerated on ECG (but not the pulse rate), the more likely HT can occur.

Hypercoagulability at AIS onset measured by INR and other tests is known to be associated with an increased thrombotic tendency. As long as the hypercoagulable state persists, both arterial and venous thromboembolic recurrences can be expected. The association of these coagulation abnormalities with HT is not always clear [30]. As we have shown here, the less the INR, the higher the risk of HT.

Having identified seven independent variables in our multivariate analysis, we applied the PP range followed by factor analysis to assigned HTI scores and to regroup HTI variables into a limited set of clusters based on shared variance. We assume probability is more intuitive for interpreting than OR. In contrast, authors of other predictive tools allocated scores based on OR changes only [5–9, 31–33]. While BLR analyzes effects of each individual predictor on the dependent variable, factor analysis isolating constructs and concepts treats the model as a whole [34]. Thus, it helped us to avoid overfitting.

Among the compared predictive tools, the SPAN-100 and HeRS were derived from the cohorts, which were very similar in clinical settings to ours (Table 13). As to the former, we have reaffirmed that it is far inferior in prognostic performance to other scores [35, 36]. Regarding the latter, the infarct size was measured in different ways: we chose the ASPECTS estimation on non-contrast CT, whereas the HeRS scored it in milliliters on magnetic resonance diffusion-weighted imaging (DWI MRI). Although both approaches are widely acceptable in hyperacute stroke settings, the ASPECTS is more suitable for non-contrast CT assessment, while the lesion volume can easily be quantified on DWI [37–39]. Furthermore, the ASPECTS, as well as the NIHSS, correlates strongly with the infarct volume (ASPECTS: Spearman ρ, −0.88; *p* < 0.001; NIHSS: Spearman ρ, 0.71; *p* < 0.001; VC, *n* = 248), but moderately with each other (Spearman ρ, −0.66; *p* < 0.001; VC, *n* = 248). If the stroke volume variable had been added to our multivariate BLR equation, a multicollinearity issue would have occurred. We also suppose that a combination of clinical and imaging features is more reliable than the imaging data alone. To make our HTI score as much easy-to-use as possible, we have purposely refrained from MRI since CT is readily available in the most hospitals. Moreover, the HeRS score is computationally complicated; therefore, it seems less attractive from the practical point of view.

There are some important peculiarities between posterior and anterior circulation stroke. The differences include the value of screening instruments, optimum diagnostic modalities, clinical features, and outcomes [40, 41]. For instance, patients with vertebrobasilar infarction have lower NIHSS score and HT rates, less often AF, higher blood glucose level and rates of false-negative DWI findings, more WBC counts, and a better long-term outcome than those with carotid stroke [42–44]. However, all aforementioned tools predict HT regardless of the vascular basin (Table 13). Moreover, scores with imaging modalities, like the HAT and SEDAN, include CT signs of MCA stroke only. Meanwhile, a scoring system, the pc-ASPECTS, has been developed and validated for posterior circulation stroke [45]. Thus, the accuracy of predictive tools could be further improved by distinguishing the infarcted vascular basins; therefore, we have decided to restrict our study to the MCA territory.

There are a few limitations in our study. A relatively small, but sufficient for statistical inferences, sample size and lack of ethnic and racial diversity could be a source of potential bias. Almost all patients came from our local community, which was populated with Russian, Tatar, and Jewish ethnic groups; there were no patients of African, Asian or Hispanic origin. Furthermore, AIS patients following endovascular interventions were excluded from the analysis due to a small number of observations. Although some clinical and imaging data were collected prospectively, the research was retrospective in nature. As a result, we were not blinded to the outcome. The study was also confined to a single clinical center; to cope with that bias, we used bootstrapping. Finally, prospective multicenter external validation would be desirable.

## Conclusions

The HTI is a four-item tool composed of ASPECTS, NIHSS, HMCA sign, and presence of AF on ECG at admission. The total score ranges from zero to eight. The higher the score, the more likely HT can occur. Knowing probability of any HT in advance could exert a significant influence on decision making to reinstitute anticoagulation in AIS patients with AF. It is a simple yet reliable instrument to predict any HT within 2 weeks after onset of AIS in the MCA territory regardless of the use of IV rtPA.

## Abbreviations

AF: Atrial fibrillation
AHT: Asymptomatic hemorrhagic transformation
AIS: Acute ischemic stroke
ALT: Alanine transaminase
APTT: Activated partial thromboplastin time
ASPECTS: Alberta Stroke Program Early CT score
AST: Aspartate transaminase
AUC: Area under the ROC curve
BCa: Bias-corrected and accelerated (adjusted for ties)
BLR: Binary logistic regression
CFA: Confirmatory factor analysis
CI: Confidence interval
CT: Computed tomography
DBP: Diastolic blood pressure
DC: Derivation cohort
DWI: Diffusion-weighted imaging
ECG: Electrocardiogram
EFA: Exploratory factor analysis
FMI: Fraction of missing information
HI-1: Hemorrhagic infarction type 1
HI-2: Hemorrhagic infarction type 2
HMCA: Hyperdense middle cerebral artery
HR: Heart rate
HT: Hemorrhagic transformation
HTI: Hemorrhagic transformation index
ICC: Intraclass correlation coefficient
ICR: Internal consistency reliability
INR: International normalized ratio
IQR: Interquartile range
IV rtPA: Intravenous recombinant tissue plasminogen activator
KDE: Kernel density estimate
LOWESS: Locally weighted scatterplot smoothing
LVEF: Left ventricular ejection fraction (echocardiography, Simpson method)
MCA: Middle cerebral artery
MCMC: Markov chain Monte Carlo
MI: Multiple imputation
MRI: Magnetic resonance imaging
NB: Normal-based
NIHSS: National Institutes of Health Stroke Scale
OR: Odds ratio
PCF: Principal component factor
PF: Principal factor
PH-1: Parenchymal hematoma type 1
PH-2: Parenchymal hematoma type 2
PP: Predicted probability
PT: Prothrombin time
RBC: Red blood cells
RE: Relative efficiency
RIV: Relative increase in variance
ROC: Receiver operating characteristic
RRR: Relative risk ratio
SBP: Systolic blood pressure
SE: Standard error
SHT: Symptomatic hemorrhagic transformation
TIA: Transient ischemic attack
VC: Validation cohort
WBC: White blood cells
